# Leprosy in the state of Amazonas: is there actually a decrease in its incidence and prevalence?^⋆^

**DOI:** 10.1016/j.abd.2021.01.007

**Published:** 2022-06-04

**Authors:** Dejanane Silva e Silva, Jamile Izan Lopes Palheta Júnior, Valderiza Lourenço Pedrosa, Carolina Talhari

**Affiliations:** a Fundação Alfredo da Matta de Dermatologia e Venereologia, Department of Tropical Dermatology, Manaus, AM, Brazil; b Curso de Pós-Graduação em Ciências Aplicadas à Dermatologia, Universidade do Estado do Amazonas, Manaus, AM, Brazil; c Universidade do Estado do Amazonas. Department of Dermatology, Manaus, AM, Brazil

Dear Editor,

According to the World Health Organization (WHO), leprosy is an important global public health problem. In 2019, 202,185 new cases were recorded, with a global prevalence of 0.22/10,000 inhabitants. India, Brazil, and Indonesia are among the countries with the highest prevalence of the disease.^1^

In Brazil, 23,612 new cases were diagnosed in 2019. In that year, a detection rate of 13.23/100,000 inhabitants was verified. In 1991, the WHO proposed the elimination of leprosy (less than one patient per 10,000 inhabitants) by 2005. Brazil did not reach this goal and has maintained a high endemicity. In 2019, among the new cases diagnosed in the country, 9.9% had grade 2 physical disability (G2D). Another important aspect, was the detection rate of new cases in patients under 15 years of age: 3.44/100,000 inhabitants. These data indicate the low quality of the leprosy control program.^1^, ^2^ Actions to control leprosy in Brazil include mainly the diagnosis of new cases during routine care at health centers, active search, and examination of contacts.^3^

In 2018, the state of Amazonas had a prevalence rate of 0.93/10,000 inhabitants. In the same year, 425 new cases were diagnosed. Despite the reduction in the detection rate from 21.54/100,000, in 2009, to 10.31/100,000, in 2018, the state has maintained a high endemicity. In 2018, 9.2% of the new patients had G2D at the diagnosis, and the detection rate of new cases in children under 15 years was 4.19/100,000.^3^

The state of Amazonas has a territorial area of 1,571,000 km^2^, divided into 62 municipalities. In 2010, the state had 3,483,985 inhabitants and the capital city, Manaus, concentrated 51.7% of the population (1,802,014 inhabitants). The Metropolitan Region of Manaus (MRM), comprising the municipalities of Autazes, Careiro, Careiro da Várzea, Iranduba, Itacoatiara, Itapiranga, Manacapuru, Manaus, Manaquiri, Novo Airão, Presidente Figueiredo, Rio Preto da Eva and Silves, had 2,210,665 inhabitants (63.4%). The state has 25 state highways, distributed mainly around the MRM.^4^, ^5^

Alfredo da Matta Foundation (FUAM, *Fundação Alfredo da Matta*), located in Manaus, is a referral center for the diagnosis and treatment of leprosy in the Amazon region. FUAM promotes regular leprosy supervision actions and the training of health professionals in the capital and other municipalities in the state. These activities are carried out by teams consisting of dermatologists, nurses, nursing and/or laboratory technicians, depending on the demand of the municipality and the need for specific training.^6^

In the present study, the results of the supervision actions carried out from March 2012 to December 2017 were assessed, with an emphasis on the detection of new cases diagnosed by FUAM teams, comparing them with the patients notified by the municipal health units.

This is a retrospective and descriptive study of new cases of leprosy diagnosed during the study period. Epidemiological data from the Notifiable Diseases Information System (SINAN, *Sistema de Informação de Agravos de Notificação*) and from the State Leprosy Coordination (*Coordenação Estadual de Hanseníase*) were used. The study was submitted to and approved by FUAM Research Ethics Committee.

All the municipalities in the state were visited by FUAM teams, some annually and others with an interval of more than one year. In 2017, supervision actions were carried out in the 62 municipalities.

During the study period, 3,299 new cases were recorded: 1,637 (49.6%) lived in the MRM and 1,662 (50.4%) in other municipalities. Of this total, 1,910 (57.9%) patients had lepromatous (multibacillary) leprosy, 303 (9.2%) had G2 at the time of diagnosis, and 362 (11%) were younger than 15 years.

During the supervision, 642 new cases were diagnosed by FUAM teams. This number is equivalent to 19.5% of the total number of patients recorded during the study period; 293 (45.6%) lived in the MRM and 349 (54.4%) in other municipalities.

Another relevant aspect of the investigation is that 1,143 (34.6%) of the new cases lived in the municipality of Manaus and among these, 808 (70.7%) were diagnosed at FUAM headquarters.

The municipalities with the highest detection rates during the study period were: Itamarati (81.25/100,000), Tapauá (76.65/100,000), Humaitá (61.05/100,000), Boca do Acre (45.25/100,000). 100,000) and Guajará (40.70/100,000; Fig. 1).

**Figure 1:**
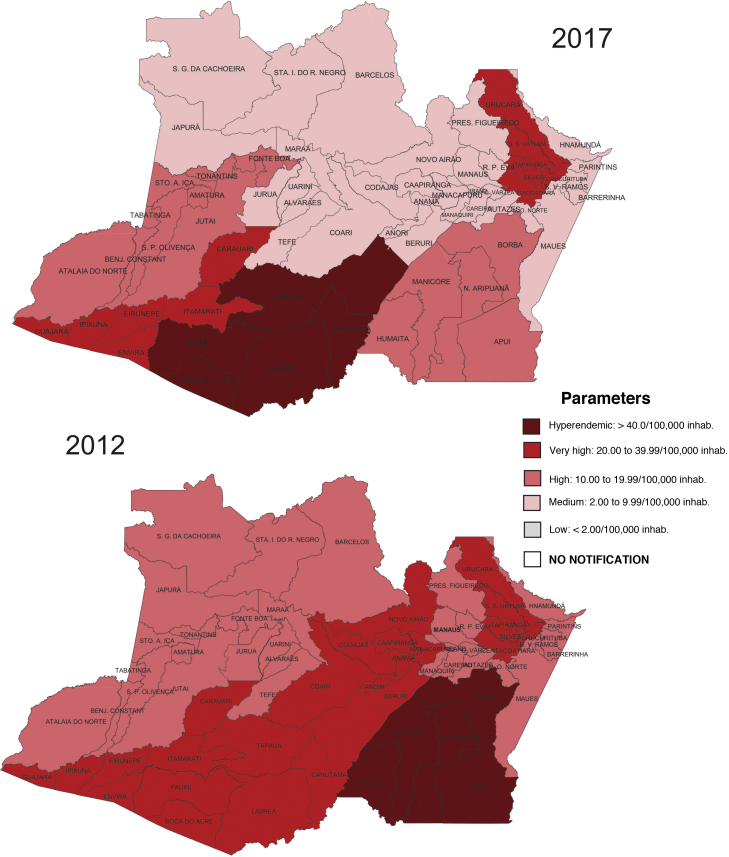
Detection rates of new leprosy cases per region of the state of Amazonas, 2012 and 2017.

In 2017, the year when all municipalities were supervised, 449 new cases of leprosy were diagnosed: 126 (28.1%) lived in the municipality of Manaus and 323 (71.9%) in the other municipalities. In that year, FUAM teams diagnosed 152 (33.8%) patients: 53 (42%) in Manaus and 99 (33.1%) in the other municipalities. Of the 126 new cases living in Manaus, 101 (80.2%) were diagnosed at FUAM headquarters.

Considering the territorial extension of the state and that most of the municipalities in the countryside lack road transportation infrastructure, it would be possible to infer that the municipalities in the MRM would have easy access to evaluation at the referral center and would notify most of the new cases. During the study period, 1,637 (49.6%) leprosy cases lived in the MRM (Table 1). However, of this total, 293 (17.9%) patients were diagnosed during the supervision actions.

**Table 1:** New cases of leprosy detected by FUAM health teams and municipal health services, from March 2012 to December 2017, in the municipalities of the metropolitan region of Manaus.

Municipalities	New cases diagnosed by the FUAM health team		New cases diagnosed by the municipality		Total
	Number of cases	Percentage	Number of cases	Percentage	
Manaus	162	14.20%	981	85.80%	1143
Presidente Figueiredo	8	17%	39	83%	47
Rio Preto da Eva	4	26.70%	11	73.30%	15
Itacoatiara	10	7.30%	127	92.70%	137
Manacapuru	12	16%	63	84%	75
Itapiranga	14	100%	0	0.0%	14
Manaquiri	4	19%	17	81%	21
Iranduba	14	26.40%	39	73.60%	53
Autazes	17	35.40%	31	67%	48
Careiro da Várzea	15	65.20%	8	34.80%	23
Careiro Castanho	11	35.50%	20	64.50%	31
Novo Airão	6	54.50%	5	45.50%	11
Silves	16	84.2%	3	15.8%	19
General total	293		1,344		1,637

The data from the municipality of Manaus are also important: of the 1,143 (34.6%) new cases diagnosed in this municipality during the study period, 808 (70.7%) were identified in FUAM. This fact indicates that patients do not seek health centers in the capital city, are not diagnosed in these places, or are systematically referred to FUAM.

When the most recent data from 2017 are analyzed, it is verified that there were no important changes during the study period. In that year, 449 new cases were recorded throughout the state: 126 (28.1%) lived in Manaus and 323 (71.9%) in other municipalities. Of this total, 152 (33.8%) cases were diagnosed during supervision actions. It was also found that more than 80% of the new cases residing in Manaus were diagnosed at FUAM.

These data explain in part the low effectiveness of the leprosy control program, with late diagnoses and a significant number of new cases in children. A late diagnosis also implies undiagnosed multibacillary cases responsible for maintenance of the endemicity.

Considering the data presented herein, showing that 642 (19.5%) new cases of leprosy would not have been diagnosed without the presence of a dermatologist, it is suggested to evaluate the activities of the Leprosy Control Program, with an emphasis on training health professionals in the capital city and municipalities, as well as enacting regular supervision actions and updates of the professionals.

## Financial support

None declared.

## Authors' contributions

Dejanane Silva e Silva: Design and planning of the study; data collection, or analysis and interpretation of data; drafting and editing of the manuscript; critical review of intellectual content; approval of the final version of the manuscript.

Jamile Izan Lopes Palheita Júnior: Design and planning of the study; analysis and interpretation of data; critical review of the intellectual content; approval of the final version of the manuscript.

Valderiza Lourenço Pedrosa: Data analysis and interpretation; critical review of the intellectual content; approval of the final version of the manuscript.

Carolina Talhari: Design and planning of the study; analysis and interpretation of data; drafting and editing of the manuscript; critical review of the intellectual content; approval of the final version of the manuscript.

## Conflicts of interest

None declared.
